# Supplementary material for: Validation of the STANDARD Q COVID-19 antigen test in Vojvodina, Serbia
Source: PLoS One. 2021 Feb 22;16(2):e0247606. doi: 10.1371/journal.pone.0247606 (PMC7899368; doi:10.1371/journal.pone.0247606)
Supplement: Attachment — Submitted filename: Reviewers Comments on the COVID-19 validation paper.docx [file pone.0247606.s001.docx]

**General Comments:**

The authors evaluated the performance of the STANDARD Q COVID-19 Ag Test among symptomatic patients who presented to health care facilities in Novi Sad, Serbia. Performance of the rapid antigen test was compared with qRT-PCR (the gold-standard diagnostic test recommended by WHO). The authors observed a strong agreement between performance of the rapid antigen test and qRT-PCR.

1. The author acknowledged the importance and novelty of such a validation in their setting and globally; however, it would have been helpful for the authors to set out a well- structured study design, with a well-defined and described study population, and selection criteria. There were major methodological weaknesses making the study design prone to a lot of biases, and hence, interpretation of the results.
2. Overall, there were many grammatical and scientific-writing errors.
3. It would have been helpful for the authors to use the standard manufacturer’s name (STANDARD Q COVID-19 Ag Test) of the rapid antigen test used for this validation.

**Specific Comments:**

1. Abstract:
a. The result and conclusion could benefit from review of grammatical and scientific-writing errors.
2. Introduction:
a. It would be helpful for the authors to review grammatical and scientific-writing errors.
b. Line 88: It would be helpful for the authors to clearly explain or edit sentence stating “no strong evidence to determine the usefulness of these tests in clinical practice”. What is being referred to as ‘strong evidence’?
c. Lines 93-94: The use of the words “potential partial replacement of qRT-PCR” appears misleading. The relevance of such a statement could be spelt out, otherwise it is debatable. Most studies have shown that the use of COVID-19 rapid antigen tests, as with many other rapid antigen tests is complementary to qRT-PCR.
3. Study design:
a. Lines 97- 99: It would be helpful for the authors to clearly state a specific study design, and to explain what is meant by “retrospective analysis of prospective collected data”. Was it a prospective or retrospective study?
b. It would be helpful for the authors to justify reasons for the short duration of the study which is a possible limitation for the generalization of the study; an inclusion of the COVID-19 prevalence would have been an added advantage in interpreting results from PPV and NPV.
c. Lines 102-104: The relevance of these sentence should be spelt out or reviewed.
d. Lines 106 -107: Inclusion and exclusion criteria should be rigid, specific and clearly stated. Information on these lines appear contrary to Line 100; Including all symptomatic patients could mean both in-patients and outpatients; COVID-19 symptoms are non-specific; hence it would have been helpful for the authors to state the criteria used by these facilities to define who a case suspected of COVID-19 is; and also, using a non-specific age group makes such a study difficult to reproduce.
e. Lines 107-109: The authors alluded that “physicians through face-face structured interviews on the day of admission interviewed all included participants”. It would be helpful for the authors to clarify what they meant by ‘day of admission’, as this sentence appears contradictory to Line 100.
f. Lines 118-119: The authors made mention of “previously trained medical staff”; the text could benefit from an explanation.
g. Lines 120-122: The authors alluded to “available tubes containing a specific viral transport medium with antifungal and antibiotic supplements”; what was the role of the antifungal and antibiotic supplements being referred to here?
h. Lines 122-126: It would be helpful for the authors to cite a reference for the laboratory protocol used.
i. Lines 128-152: Cite reference(s); this text could be shortened.
j. Lines 156-157: Cite reference(s).
4. Data Analysis:
a. Lines 174-176: The relevance of the broad classification of statistical analyses used should be spelt out, otherwise it would be helpful for the authors to indicate specific statistical analyses used.
b. Lines 177-178: The authors should review the scientific-writing of statistical tests. For example, it is commonly written Kruskal Wallis test or Kruskal-Wallis ANOVA rather than ANOVA-Kruskal±Wallis H-test; and Fisher’s Exact test rather than Fisher exact test. It would be helpful for the authors to also indicate that Kruskal-Wallis ANOVA was used for ordinal scale variables.
c. Lines 184-186: It would be helpful for the authors to clarify whether the accuracy and Kappa values used were derived from validation done by the manufacturer.
d. How was sample size determined?
e. Any consideration for missing data?
5. Ethical Considerations:
a. Lines 190-191: Why was verbal informed consent obtained only at the moment of sample collection? It would be helpful for the authors to explain why informed consent was not sought for the overall conduct of the research which also include, but not limited to data and sample collection.
6. Results:
a. It would be helpful for the authors to review the scientific-writing in this section.
b. Line 196: The relevance of the information should be spelt out as this sentence appears contrary to Line 100 stating only outpatients were enrolled in the study.
c. Lines 196-198: The relevance of the information should be spelt out, otherwise its inclusion is questionable.
d. It would be helpful for the authors to explain why fever was not measured.
e. Lines 201: The relevance of the information in line should be spelt out.
f. Lines 203-204: It would be helpful for the authors to explain the relevance of both a mean period of 9.4 days and a median time of 5.
g. Lines 206-207: The relevance of the information in these lines should be spelt out.
h. Lines 225-227: It would be helpful for the authors to explain the relevance of the values in the brackets.
i. What is the overall observed period in Line 229 referring to?
7. Discussion:
a. It would be helpful for the authors to review the grammar and scientific-writing in the section, and by extension interpretation of the results.
b. Line 257: The relevance of the information could be spelt out, otherwise its inclusion in this section is debatable
c. Lines 266-268: These lines appear contradictory to Lines 107-109 and Line 196. Were patients who presented with symptoms requiring admission excluded because they it was an exclusion criterion, or no patient with symptoms needed admission?
d. Lines 271-272: These lines appear contradictory to Line 106 stating all symptomatic patients were included. It would be helpful for the authors to clarify.
e. Cite reference(s) for Lines 304-306.
f. Lines 324: Cite reference stating the prevalence of positivity
g. Was there an explanation for participants presenting to facilities and being tested after ≥ 16 days from onset of signs/symptoms?
8. Tables:
a. Table 1: It would be helpful for the authors to consider using figures for “days from symptoms onset”; for example, 0-5 days for the “first five days”.
b. Line 215: It would be helpful for the authors to differentiate P values derived from Fisher’s Exact test or chi-square test
c. Table 2: It would be helpful for the authors to explain the meaning of NA underneath the table;
d. Table 2: Lines 239-240: The title of the table does not reflect the findings; the authors should consider replacing “Similarities and differences” with “Comparison”.
9. Figures:
a. Figure 2: The authors should consider indicating what unit is the period between symptom onset and laboratory testing on the Y-axis measured in?
