# Supplementary material for: Validation of the STANDARD Q COVID-19 antigen test in Vojvodina, Serbia
Source: PLoS One. 2021 Feb 22;16(2):e0247606. doi: 10.1371/journal.pone.0247606 (PMC7899368; doi:10.1371/journal.pone.0247606)
Supplement: Attachment — Submitted filename: General Comments 2nd Review.docx [file pone.0247606.s003.docx]

General Comments:

The authors evaluated the performance of the STANDARD Q COVID-19 Ag Test (a rapid antigen test) against qRT-PCR (the gold-standard laboratory diagnostic test for SARS-CoV2 as recommended by WHO) among symptomatic patients suspected of Covid-19, who presented to primary and tertiary health care facilities in Novi Sad, Serbia. The authors observed a strong agreement between performance of the rapid antigen test and qRT-PCR.

The authors acknowledged the importance, timing and novelty of such a validation in their setting and globally. Significant changes were made to the manuscript from previous review. However, this manuscript could benefit from further review for scientific-writing errors. Examples include, but not limited to:

1. Repetition of words with resulting long text – see Lines 85,117, 143, 284 etc.

2. Slightly confusing presentation of results and discussion section – See Lines 212 -214, 217 – 220, 238-241, 257 (which of the two statistical tests was used, or both were used and what is the possible explanation for that), 314 – 318 etc.

The study could also benefit from a rigid study design for ease of reproducibility, reduction of bias and generalizability. It would be helpful for the authors to briefly explain how the sample size of 120 was determined. Was this a pre-specified/ determined sample size? The statement in Line 109 should be clarified. Asymptomatic patients were excluded from which “analysis” or were they pre-specified to be excluded from the study as stated in Line 352. Based on what should close contacts (could be asymptomatic or symptomatic) of index cases in families and “collectives” as stated Lines 340- 343 be tested using the RDT, when the study population was only symptomatic patients? It would have been helpful for the authors to also cite reference(s), criteria or case definitions used for the determination of the study population (i.e., patients with mild and moderate signs and symptoms).
